# Supplementary material for: Structure of SALL4 zinc finger domain reveals link between AT-rich DNA binding and Okihiro syndrome
Source: Life Sci Alliance. 2023 Jan 12;6(3):e202201588. doi: 10.26508/lsa.202201588 (PMC9838217; doi:10.26508/lsa.202201588)
Supplement: Supplementary file 2 [file LSA-2022-01588_TableS1.docx]

**Table S1. Variants of uncertain significance**

| **Missense mutation** | **ClinVar accession/Reference** |
| --- | --- |
| I84V | 655640 |
| C220Y | 1334823 |
| L226P | 423383 |
| K381R | 1306787 |
| P409R | 593400 |
| R418H | 218770 |
| K475R | 931969 |
| R601P | 1507502 |
| R890W | 850032 |
| G911D | https://onlinelibrary.wiley.com/doi/full/10.1002/ajmg.a.37066 |
| S950K | 423567 |
| V995G | 1370536 |
