# Supplementary material for: Structure of SALL4 zinc finger domain reveals link between AT-rich DNA binding and Okihiro syndrome
Source: Life Sci Alliance. 2023 Jan 12;6(3):e202201588. doi: 10.26508/lsa.202201588 (PMC9838217; doi:10.26508/lsa.202201588)
Supplement: Supplementary file 3 [file LSA-2022-01588_TableS2.docx]

**Table S2. Data collection and structural refinement statistics**

| Data collection | SALL4-pall01 complex 8A4I |
| --- | --- |
| Source, wavelength | DLS I04, 1.28216 Å |
| Number of crystals | 1 |
| Space group | *P*1 |
| Cell parameters a, b, c (Å) | 39.03 66.11 77.94 |
| α, β, γ (°) | 73.04 76.43 76.14 |
| Resolution (Å) | 73.4-2.8 (3.3-2.78) |
| Multiplicity | 3.3 (3.1) |
| Average I/σ(I) | 3.1 (1.9) |
| Completeness (ellipsoidal) (%) | 0.77 (0.31) |
| R_merge_ | 0.45 (0.91) |
| R_meas_ | 0.54 (1.10) |
| R_pim_ | 0.29 (0.62) |
| CC_1/2_ | 0.67 (0.89) |
| Wilson B factor (Å^2^) | 45 |
| Refinement |  |
| Unique reflections used for refinement | 6179 |
| R_work_/R_free_ (%) | 24.7 /25.4 |
| Average B factors (Å^2^) | 72 Å^2^ |
| R.m.s. deviations |  |
| Bond lengths (Å) | 0.006 |
| Bond angles (°) | 0.83 |
| Ramachandran |  |
| Favoured (%) | 95 |
| Additionally allowed (%) | 5 |
| Outliers (%) | 0 |
