# Supplementary material for: Structure of SALL4 zinc finger domain reveals link between AT-rich DNA binding and Okihiro syndrome
Source: Life Sci Alliance. 2023 Jan 12;6(3):e202201588. doi: 10.26508/lsa.202201588 (PMC9838217; doi:10.26508/lsa.202201588)
Supplement: Supplementary file 5 [file LSA-2022-01588_TableS3.docx]

**Table S3 – SAXS experimental details and data parameters**

| (*a*) Sample details ZFC4 DNA ZFC4-DNA | | | |
| --- | --- | --- | --- |
| Organism | *M. musculus* | - | Combination of ZFC4 and DNA |
| Source (Catalogue No. or reference) | *E. coli* expressed | IDT DNA oligos | Combination of ZFC4 and DNA |
| Uniprot ID (residues in construct) + uncleaved tag | Q8BX22 870-940  +3C scar (GPDS) at N-terminus | 5’CATATTAATATC3’  3’GTATAATTATAG5’ | Combination of ZFC4 and DNA |
| Extinction coefficient ε (A_280_, 0.1%(w/v) | 0.210 | - | - |
| Molecular mass *M* from chemical composition (Da) | 8426 | 7446 | 15872 (assuming 1:1 binding) |
| SEC-SAXS column, s200 increase 3.2/200  Loading concentration (mg/ml)  Injection volume (µl)  Flow rate (ml/min) | 6.69  45  0.1 | 5.00  45  0.1 | 5.80  45  0.1 |
| Concentration measurement method | BCA assay | - | - |
| Solvent composition | 20 mM Tris pH 7.5 at 4°C, 200 mM NaCl | | |
| (*b*) SAS data collection parameters | | | |
| Instrument Diamond Light Source Ltd Synchrotron. BL21 beamline, Eiger 4M detector (Dectris) | | | |
| Wavelength (Å) 0.9998 | | | |
| Beam size at focal point (µm) 34x40  Sample-to-detector distance (m) 4.014 | | | |
| *q*-measurement range (Å^-1^) 0.0026 – 0.34 | | | |
| Exposure time 3 s | | | |
| Sample temperature (ºC) 22 | | | |
| (*c*) Software employed for SAS data reduction, analysis and interpretation | | | |
| Sample – Solvent subtraction Chromixs from ATSAS 3.0.5 (Manalastas-Cantos et al., 2021) | | | |
| Calculation of ε from sequence ProtParam (Gasteiger et al., 2005) | | | |
| Basic analyses: Guinier, *P*(*r*), *V*_P_ PRIMUS from ATSAS 3.0.5 (Manalastas-Cantos et al., 2021) | | | |
| Shape/bead modelling DAMMIF from ATSAS 3.0.5 (Manalastas-Cantos et al., 2021) | | | |
| Crysol from PRIMUS in ATSAS 3.0.5 (Manalastas-Cantos et al., 2021) | | | |
| Modelling of missing sequence COOT (Emsley and Cowtan, 2004) | | | |
| Molecular graphics PyMOL 2.4.1 and ChimeraX 1.3 | | | |
| (*d*) Structural parameters ZFC4 DNA ZFC4-DNA | | | |
| Guinier Analysis |  |  |  |
| *I*(0) (cm^-1^) | 0.082 ± 0.00005 | 0.014 ± 0.000069 | 0.0075 ± 0.000077 |
| *R*_g_ (Å) | 20.77 ± 0.24 | 16.72 ± 0.17 | 18.23 ± 0.36 |
| q range (Å^-1^) | 0.0026 – 0.340 | 0.0026 – 0.340 | 0.0026 – 0.340 |
| Quality of Fit χ^2^ value (p-value) | 1.24 (0.00692) | 1.08 (0.198) | 0.857 (0.953) |
| *P*(*r*) analysis |  |  |  |
| *I*(0) (cm^-1^) | 0.0079 ± 0.0000394 | 0.0133 ± 0.0000536 | 0.00719 ± 0.0000608 |
| *R*_g_ (Å) | 20.08 ± 0.0819 | 16.02 ± 0.0634 | 17.48 ± 0.124 |
| *d*_max_ (Å) | 58.22 | 45.82 | 50.56 |
| *q-*range (Å^-1^) | 0.00412 – 0.340 | 0.00487 – 0.340 | 0.00675 – 0.340 |
| Quality of Fit χ^2^ value (p-value) | 1.01 (0.351) | 1.03 (0.124) | 1.01 (0.322) |
| Porod volume (Å^-3^) | 8564.57 | 12052.90 | 14565.90 |
| (*e*) Shape modelling results ZFC4 DNA ZFC4-DNA | | | |
| DAMMIF (default parameters, 10 repetitions), averaged with DAMAVER and refined with DAMMIN | | | |
| *q-*range for fitting | 0.00412 – 0.340 | 0.00487 – 0.340 | 0.00675 – 0.340 |
| Symmetry/anisotropy assumptions | P1, none | P1, none | P1, none |
| NSD (standard deviation) | 0.753 (0.044) | 0.752 (0.046) | 0.754 (0.055) |
| χ^2^ value | 0.9915 | 1.032 | 1.013 |
| Constant subtraction procedure | Skipped | 0.0001656 | 0.0001076 |
| Model resolution (from SASRES) (Å) | 20 | 17 | 20 |
| (*f*) Atomistic modelling ZFC4 DNA ZFC4-DNA | | | |
|  |  |  |  |
| CRYSOL (with default parameters) |  |  |  |
| *q-*range for fitting |  |  |  |
| χ^2^ value (p-value) | 1.012 (0.333) | 1.272 (1.58x10^-19^) | 1.145 (3.35x10^-7^) |
| Predicted Rg (Å) | 21.56 | 14.28 | 16.88 |
| Vol (Å), Ra (Å), Dro (e Å^-3^) | 10008, 1.400, 0.068 | 7717, 1.400, 0.075 | 17725, 1.800, 0.000 |
| (*g*) Data and model deposition IDs | SASDP64 | SASDP74 | SASDP84 |
|  |  |  |  |
|  |  |  |  |

Manalastas-Cantos, K., Konarev, P.V., Hajizadeh, N.R., Kikhney, A.G., Petoukhov, M.V., Molodenskiy, D.S., Panjkovich, A., Mertens, H.D.T., Gruzinov, A., Borges, C., Jeffries, C.M., Svergun, D.I., Franke, D. (2021)
[ATSAS 3.0: expanded functionality and new tools for small-angle scattering data analysis](https://journals.iucr.org/j/issues/2021/01/00/ge5081/index.html) *J. Appl. Cryst.* 54, 343-355 [DOI](https://doi.org/10.1107/S1600576720013412)

Gasteiger E., Hoogland C., Gattiker A., Duvaud S., Wilkins M.R., Appel R.D., Bairoch A.;
*Protein Identification and Analysis Tools on the ExPASy Server;*
(In) [John M. Walker (ed): The Proteomics Protocols Handbook, Humana Press (2005)](http://www.springer.com/life+sciences/biochemistry+%26+biophysics/book/978-1-58829-343-5).
pp. 571-607

Emsley, P. and K. Cowtan (2004). "Coot: model-building tools for molecular graphics." Acta Crystallogr D Biol Crystallogr 60(Pt 12 Pt 1): 2126-2132.
