# Supplementary material for: Structure of SALL4 zinc finger domain reveals link between AT-rich DNA binding and Okihiro syndrome
Source: Life Sci Alliance. 2023 Jan 12;6(3):e202201588. doi: 10.26508/lsa.202201588 (PMC9838217; doi:10.26508/lsa.202201588)

Figure 3 EMSA replicate 1

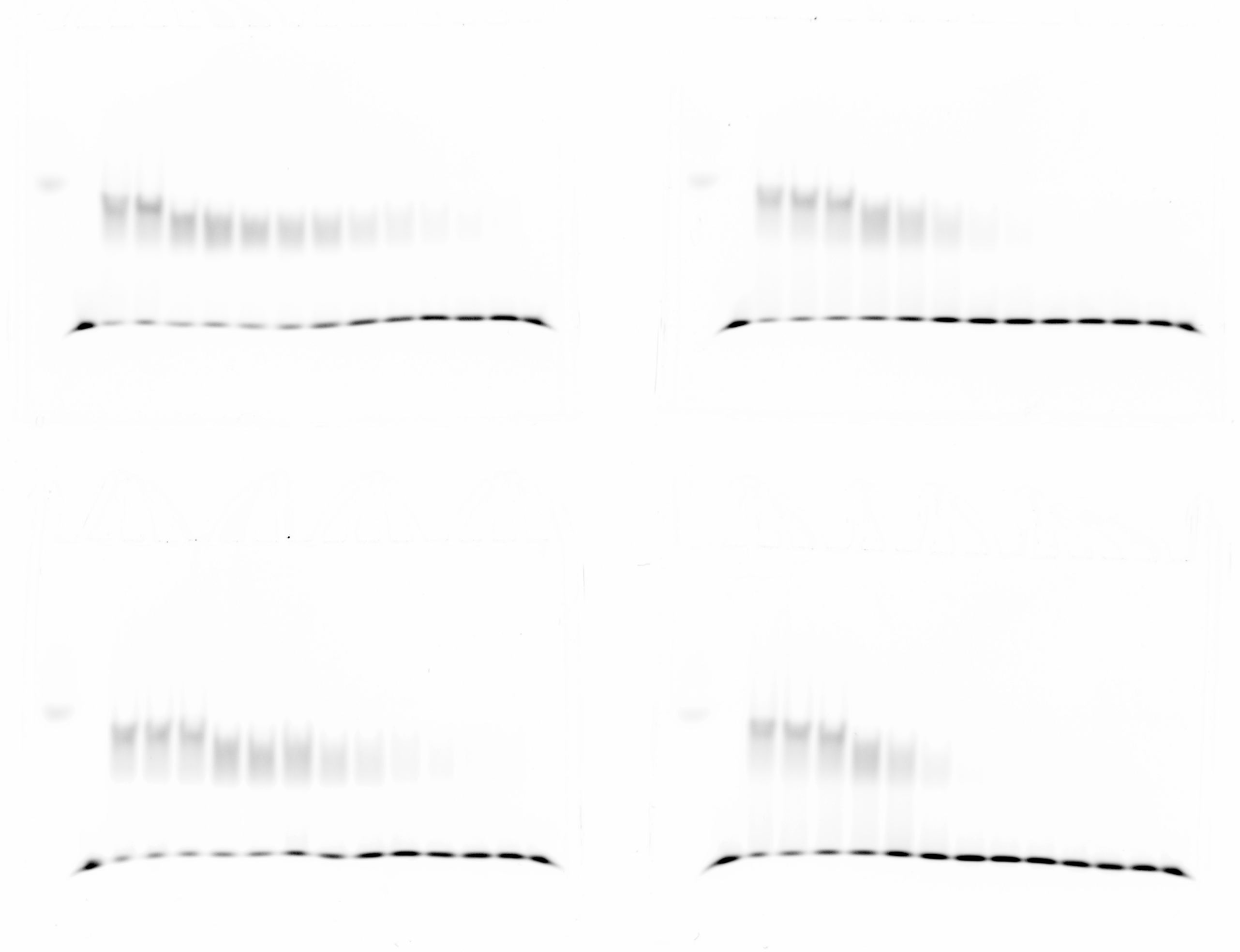

Figure 3 EMSA replicate 2

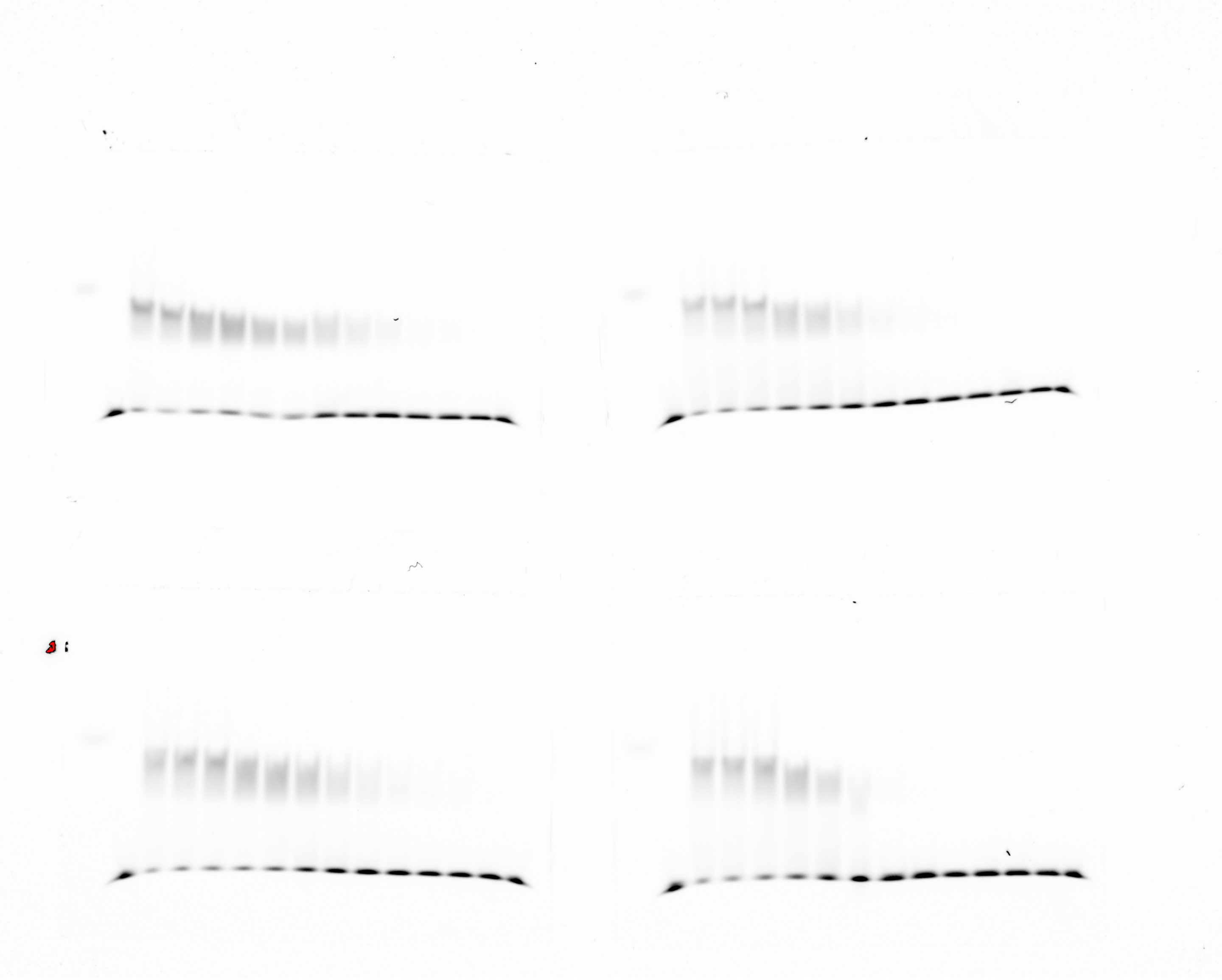

Figure 3 EMSA replicate 3

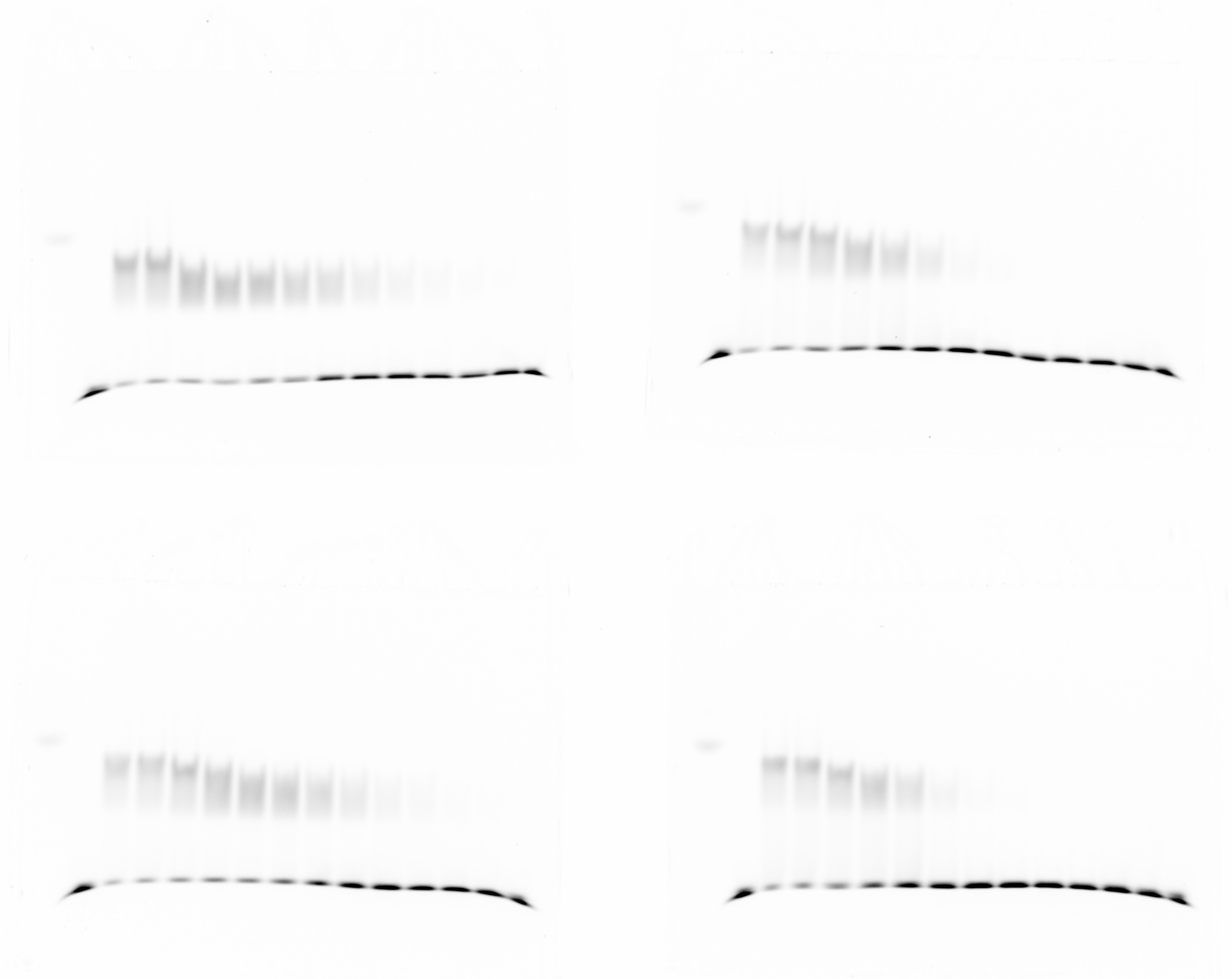

Figure 4 EMSA replicate 1

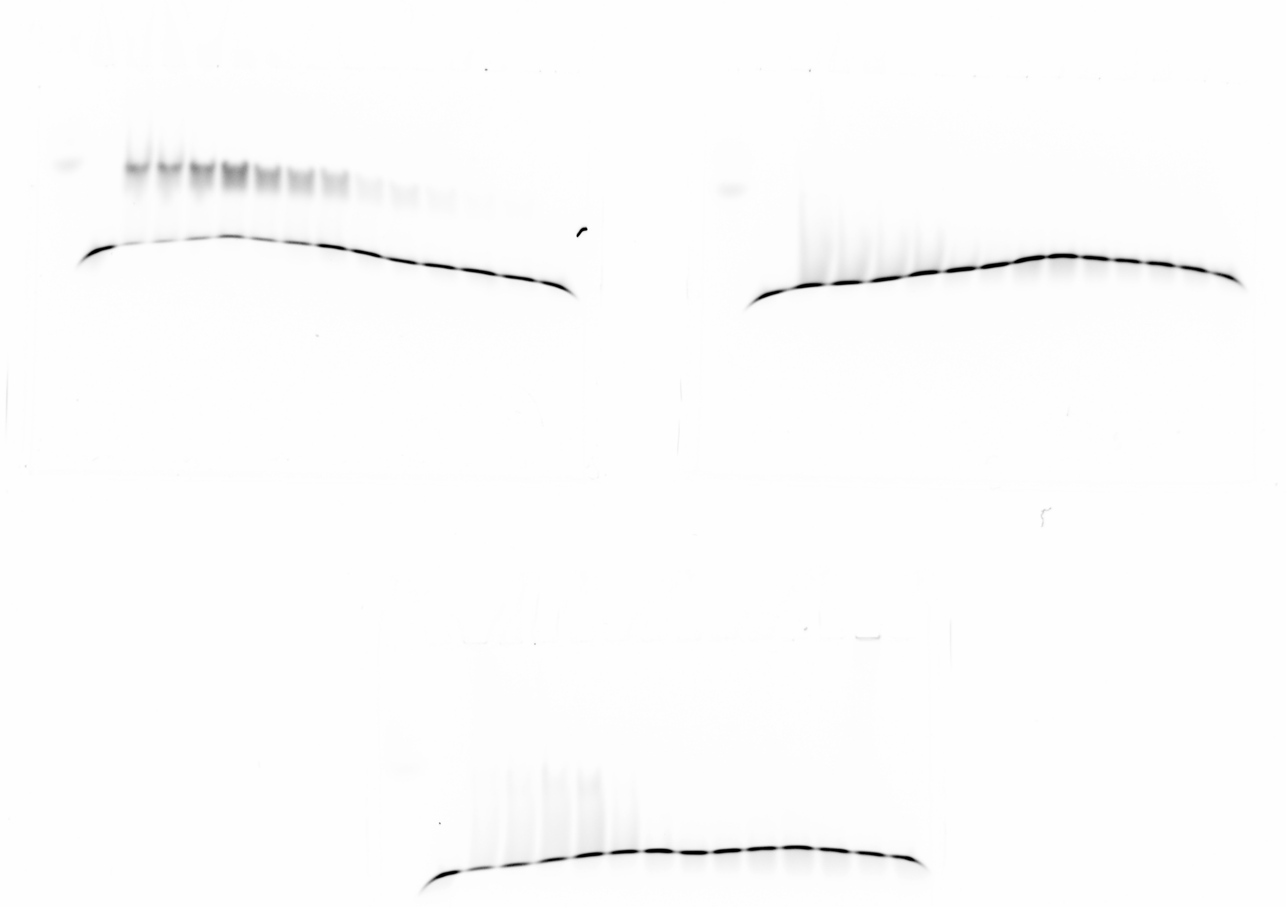

Figure 4 EMSA replicate 2

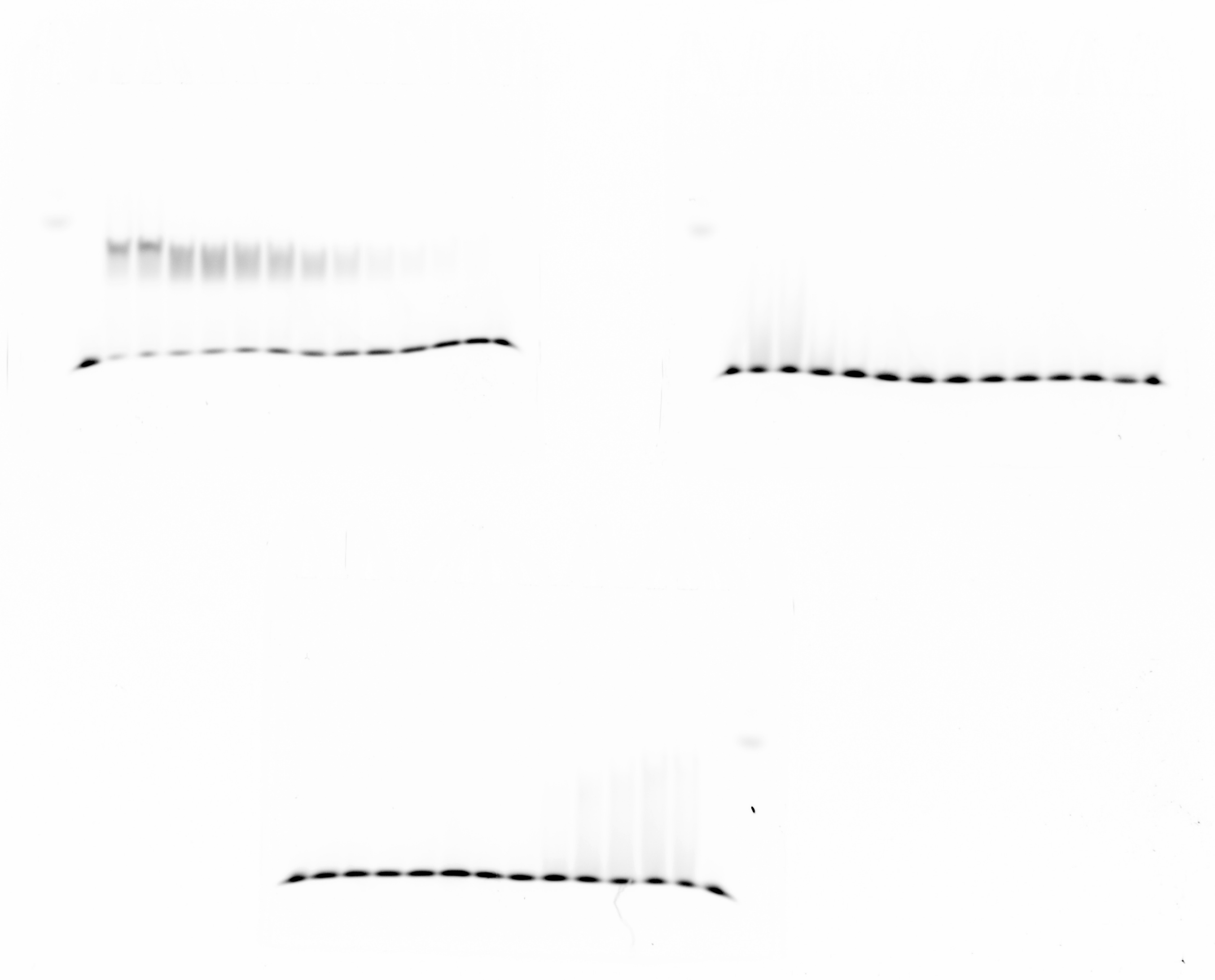

Figure 4 EMSA replicate 3

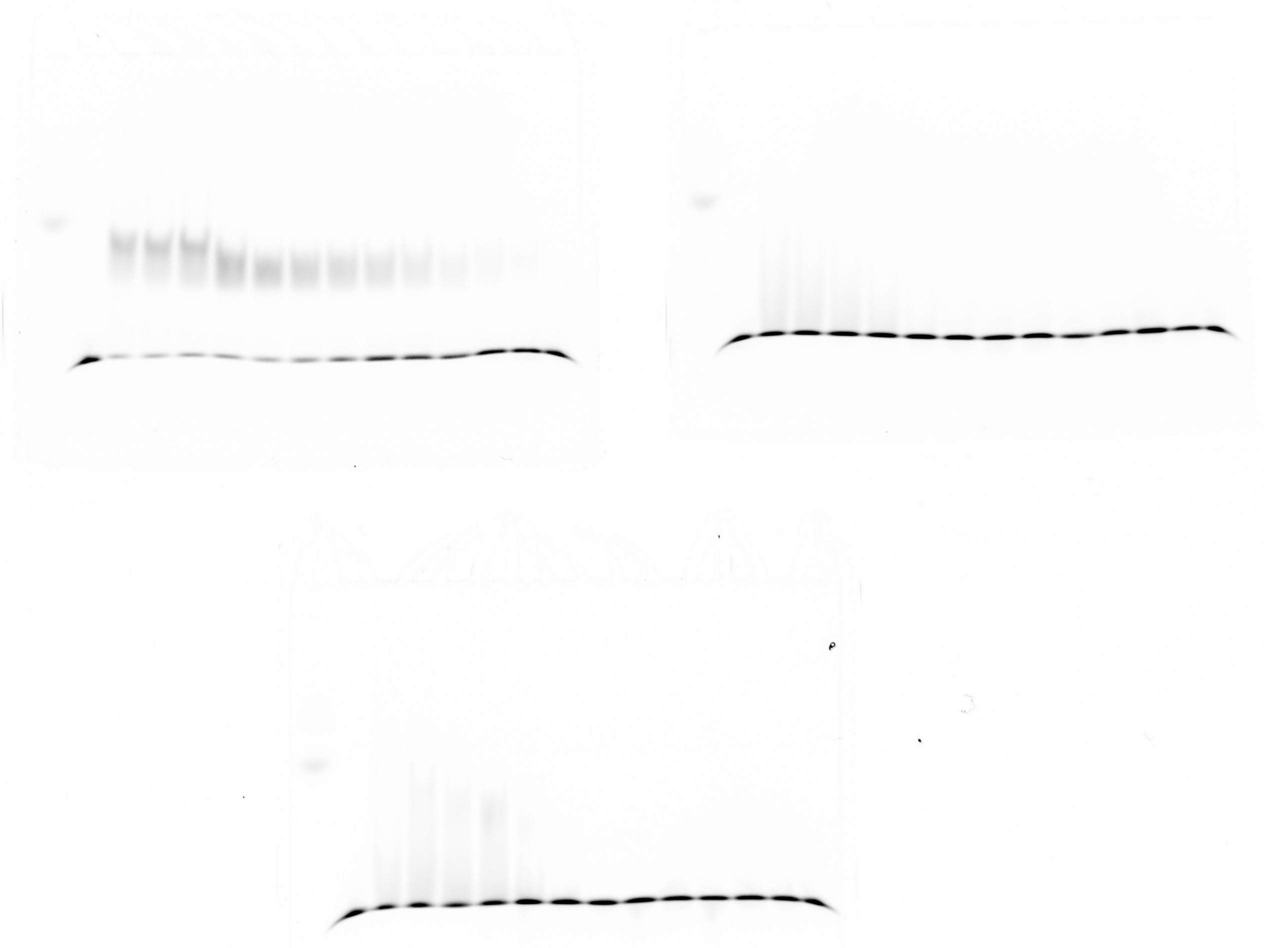

Coomassie gel of purified proteins

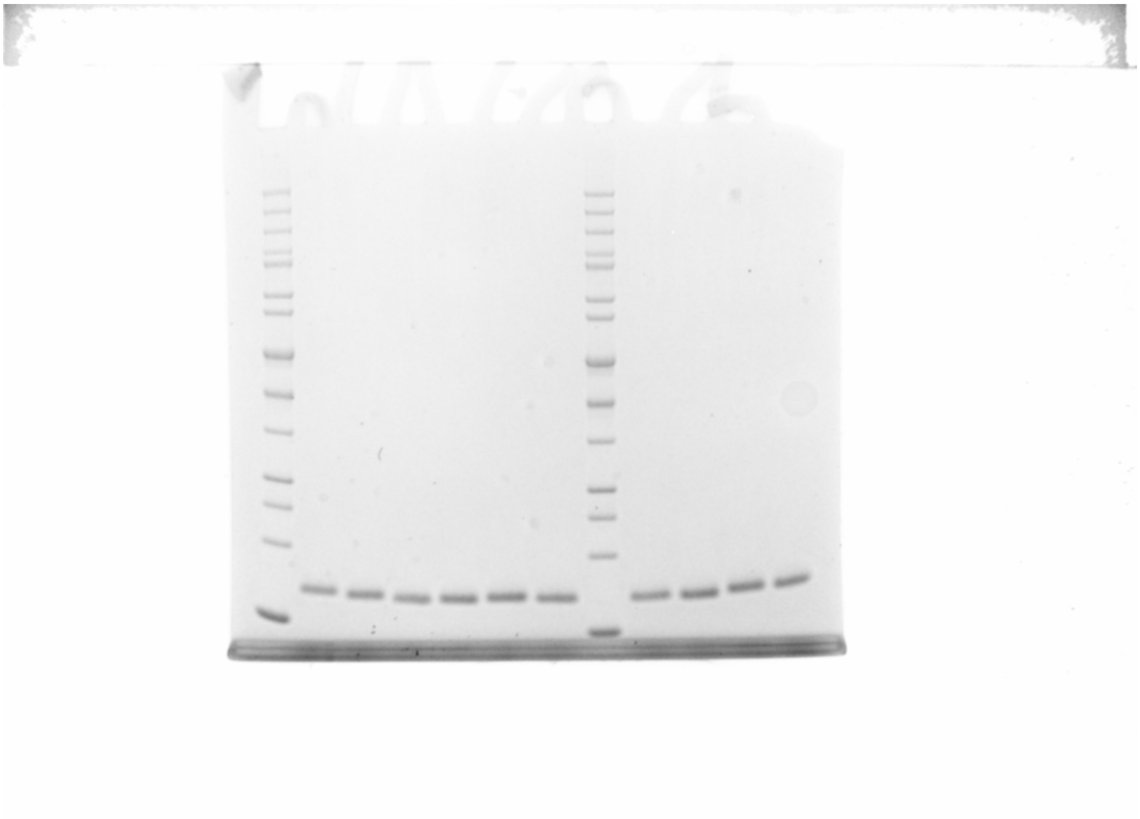

Supplement: Supplementary file 9 [file LSA-2022-01588_SdataF3.3_F4.3.pdf]
