# Supplementary material for: Structure of SALL4 zinc finger domain reveals link between AT-rich DNA binding and Okihiro syndrome
Source: Life Sci Alliance. 2023 Jan 12;6(3):e202201588. doi: 10.26508/lsa.202201588 (PMC9838217; doi:10.26508/lsa.202201588)
Supplement: Supplementary file 11 [file LSA-2022-01588_TableS4.docx]

**Table S4. Dissociation constant calculations**

| **EMSA dataset** | **Sample** | **Kd (µM)** | **95% confidence interval (µM)** | **R^2^** |
| --- | --- | --- | --- | --- |
| Major groove binding surface mutations | WT | 0.76 | 0.55 - 1.0 | 0.9322 |
|  | I897S | 4.8 | 3.7 - 6.3 | 0.9608 |
|  | V925S | 0.91 | 0.70 - 1.2 | 0.9546 |
|  | I897S/V925S | 5.1 | 3.8 - 6.8 | 0.9527 |
| Patient mutations | WT | 0.47 | 0.37 - 0.61 | 0.9621 |
|  | R900W | 23 | 13 - 50 | 0.8835 |
|  | G921D | ND | ND | ND |
