# Supplementary material for: Structure of SALL4 zinc finger domain reveals link between AT-rich DNA binding and Okihiro syndrome
Source: Life Sci Alliance. 2023 Jan 12;6(3):e202201588. doi: 10.26508/lsa.202201588 (PMC9838217; doi:10.26508/lsa.202201588)
Supplement: Supplementary file 16 [file LSA-2022-01588_TableS5.docx]

**Table S5 Antibody dilutions for immunofluorescence**

| **Target** | **Application** | **Product reference** | **Working dilution** |
| --- | --- | --- | --- |
| SALL4 | Immunofluorescence | Abcam cat. ab29112 | 1:200 |
| SALL4 | Immunofluorescence | Santa Cruz cat. sc-101147 | 1:50 |
| SALL1 | Immunofluorescence | Abcam cat. ab41974 | 1:200 |
